# Rhomboid antigens are promising targets in the vaccine development against *Toxoplasma gondii*

**DOI:** 10.17179/excli2018-1993

**Published:** 2019-05-21

**Authors:** Masoud Foroutan, Leila Zaki, Sanaz Tavakoli, Shahrzad Soltani, Ali Taghipour, Fatemeh Ghaffarifar

**Affiliations:** 1 Abadan School of Medical Sciences, Abadan, Iran; 2 Department of Parasitology, Faculty of Medical Sciences, Tarbiat Modares University, Tehran, Iran; 3 Department of Parasitology and Mycology, School of Medicine, Isfahan University of Medical Sciences, Isfahan, Iran

**Keywords:** Toxoplasma gondii, rhomboid, vaccines, immunization, adjuvant

## Abstract

*Toxoplasma gondii* (*T. gondii*) is an obligate intracellular parasite with worldwide distribution. It is estimated that near one-third of the people around the globe are latently seropositive for the parasite. Since the current common drugs are incapable in the elimination of parasites within tissue cysts, the development of an effective vaccine has high priority for researchers to limit the infection. During recent years, non-stop efforts of scientists have made great progress in the identification and development of *T. gondii* candidate vaccines. However, there is a lack of a commercially licensed vaccine for human application yet. Rhomboid proteases (ROMs) are a class of serine proteases that have an important role in the invasion of the parasites that can be considered as a new target for vaccine strategy. They also play critical roles in mitochondrial fusion and growth factor signaling, allowing the parasite to completely enter into the host cell. In the current review, we have summarized the recent progress regarding the development of ROM-based vaccines against acute and chronic *T. gondii* infection in animal models.

## Introduction

*Toxoplasma gondii* (*T. gondii*) is an obligate intracellular protozoan with cosmopolitan distribution in five continents (Foroutan-Rad et al., 2016[19]; Wang et al., 2017[49]) which can infect several warm-blooded vertebrates such as rodents, birds, marine mammals, domestic/wild mammals, etc. (Dubey, 2008[12]; Khademvatan et al., 2017[28]; Rostami et al., 2017[40]). Moreover, *T. gondii* has been isolated from Iranian snakes (Nasiri et al., 2016[37]). It is estimated that more than one-third of the human population are latently infected with the parasite in different groups throughout the globe (Foroutan-Rad et al., 2016[19]; Wang et al., 2017[49]). In general, toxoplasmosis is oftentimes asymptomatic among immunocompetent subjects, whereas in organ transplant recipients, HIV-positive persons, individuals with malignancy, and newborns can cause severe complications and life-threatening outcomes (Fallahi et al., 2018[14]; Gharavi et al., 2011[22]; Soltani et al., 2018[44]; Wang et al., 2017[49]).

More recently, based on systematic review and meta-analysis papers from a global perspective, the positive association between toxoplasmosis with some diseases (type 2 diabetes mellitus: odds ratio [OR]: 2.39; *P* = 0.013) and psychiatric disorders such as bipolar disorder (OR: 1.52; *P* = 0.02), obsessive-compulsive disorder (OR: 3.4; *P* < 0.001), and schizophrenia (OR: 1.81; *P* < 0.00001) have been documented (Majidiani et al., 2016[34]; Sutterland et al., 2015[45]).

The commonly prescribed drugs for the treatment of *T. gondii* infection only can limit the multiplication of tachyzoites in the initial phase of infection, while they are unable to eliminate the encysted parasites within tissue cysts (Antczak et al., 2016[1]). Accordingly, the development of an effective vaccine has high priority for investigators to limit the infection in animals and humans. During the three past decades, non-stop efforts of researchers have made great achievements in the identification and development of *T. gondii* candidate vaccines mainly on rhoptry proteins (ROPs), surface antigens (SAGs), microneme proteins (MICs), dense granule antigens (GRAs), calcium-dependent protein kinases (CDPKs), and some other antigens (Foroutan and Ghaffarifar, 2018[15]; Foroutan et al., 2018[18], 2019[16]; Kur et al., 2009[30]; Zhang et al., 2015[50]).

In the past several years, the different vaccine types with various strategies have been developed experimentally throughout the globe that ranged from killed vaccines, live or attenuated vaccines, recombinant subunit proteins, live vectors to DNA vaccines or multiepitope-based vaccines (Foroutan and Ghaffarifar, 2018[15]; Foroutan et al., 2019[16]; Ghaffarifar, 2015[21], 2018[20]; Kur et al., 2009[30]; Zhang et al., 2015[50]). These vaccines with various vaccination routes in different mouse models have verified partial protection and reduction of brain cysts burden after challenge with avirulent and/or virulent *Toxoplasma* strains (Foroutan and Ghaffarifar, 2018[15]; Foroutan et al., 2018[18]; Han et al., 2017[24]; Kur et al., 2009[30]; Li et al., 2012[32]; Zhang et al., 2015[50]; Zhang et al., 2015[51]). However, currently there is a lack of an approved commercial vaccine for human use (Zhang et al., 2015[50]).

## Rhomboid Proteases (ROMs)

In apicomplexan protozoa, including *Toxoplasma* and *Plasmodium*, a number of specific molecules are required for entering into cells or invasion procedure (Dowse et al., 2008[9]). Rhomboid proteases are a class of serine proteases that have an important role in the invasion of the parasites (Kim, 2004[29]). Invasion by *T. gondii* and generally all apicomplexan parasites is carried out by adhesion receptors. Rhomboid proteases are thought to cleave adhesins within their transmembrane segments, thus allowing the parasite to disengage from receptors and completely enter the host cell (Shen et al., 2014[43]). ROM antigens are conserved molecules in *T. gondii* which recently become attractive as a target in vaccine based purposes (Dowse et al., 2005[10]; Li et al., 2012[32]). These ubiquitous serine proteases are able to recognize and cleave their substrates within their transmembrane domains. Recent data strongly suggest that parasite-derived, rhomboid like proteases cleave MIC proteins in order to release them from the parasite membrane (Brossier et al., 2008[3]). They also play critical roles in mitochondrial fusion, growth factor signaling, and invasion procedure (Li et al., 2012[32]). 

In *Plasmodium* spp, they have been named PfROM1, PfROM3, PfROM4 and PfROM6 (Kim, 2004[29]). In *Theileria* *annulata* and *T. parva*, only ROM4 and ROM6 have been found (Dowse and Soldati, 2005[11]). The nomenclature is carried out using the first letters of the genus and species and using ROM and the number of each rhomboid. Every number shows the order of its detection (Dowse and Soldati, 2005[11]). For the first time, ROMs have been identified in *Drosophila melanogaster*, where they have a signaling role (Brossier et al., 2008[3]), it also has a cleaving role (Brossier et al., 2005[2]). *T. gondii* possesses six ROMs namely TgROM1-TgROM6 which are expressed in different stages of the life cycle (Dowse et al., 2005[10]). For instance, TgROM1 is localized to the micronemes and Golgi and participates in the invasion process. Since the position of ROM1 is in the micronemes, so it acts only during invasion not before it (Dowse et al., 2008[9]). TgROM2 and TgROM3 are localized to the Golgi and mainly are expressed in sporozoites (Brossier et al., 2008[3]; Dowse et al., 2008[9]; Shen et al., 2014[43]). TgROM4 exits in plasma membrane (Shen et al., 2014[43]), and is expressed mainly in tachyzoites, and to a less extent in bradyzoites and sporozoites (Han et al., 2017[24]). ROM4 is an activator for the micronemes and micronemes activity depends on ROM4. Also, ROM4 counts as the first molecule in invasion (Rahimi et al., 2017[38]; Shen et al., 2014[43]). TgROM4 is the central molecule in regulating MIC2, MIC3, and MIC6 and helps the parasite to enter into cells (Han et al., 2017[24]). TgROM5 localizes to the surface at the posterior end and is the functioning ROM against a wide number of substrates unlike other ROMs (Kim, 2004[29]).

In a study, Brossier et al. investigated the role of microneme rhomboid protease TgROM1 during *in vitro* growth of *T. gondii*. The results showed that suppression of TgROM1 using the tetracycline-regulatable system caused a modest decrease in invasion role of TgROM1 and also ROM1 is essential for appropriate intracellular growth of *T. gondii* (Brossier et al., 2008[3]). Also, in 2010 Buguliskis et al. investigated the role of TgROM4 using a knockout technique, the results showed that suppression of TgROM4 led to interruption of normal gliding and cell motility and finally invasion process. Therefore, TgROM4 is critical for efficient cell motility and invasion of host cells by *T. gondii* (Buguliskis et al., 2010[4]). Furthermore, Shen et al. (2014[43]) carried out a single, double, and triple knockout for the three ROMs expressed in *T. gondii* tachyzoites to demonstrate the exact roles of each ROM during the invasion. The results revealed that ROM4 acts first in adhesin processing and host cell invasion, whereas ROM1 or ROM5 do not have a prominent role in the invasion (Shen et al., 2014[43]). The specific features and main functions of some ROMs have been inserted in Table 1(Tab. 1) (References in Table 1: Brossier et al., 2005[2]; Brossier et al., 2008[3]; Buguliskis et al., 2010[4]; Dowse and Soldati, 2004[8]; Dowse and Soldati, 2005[11]; Dowse et al., 2005[10]; Dowse et al., 2008[9]; Han et al., 2017[24]; Kim, 2004[29]; Li et al., 2012[32]; Rugarabamu et al., 2015[41]; Shen et al., 2014[43]; Zhang et al., 2015[51]).

## DNA Vaccines

The DNA vaccination as a novel strategy has a critical role against acute and chronic *T. gondii* infection; it has very strong immunogenicity and can induce both humoral and cellular immunity in the body as well as stimulates dendritic cells (DCs) to be matured and makes them strong stimulators of T-cell immunity. Therefore, in studies of vaccines against T. gondii infection, the DNA vaccination has received much attention (Foroutan et al., 2019[16]; Kur et al., 2009[30]; Li and Petrovsky, 2016[33]; Zhang et al., 2015[50]). Some advantages of DNA vaccines compared with traditional vaccines are as follows: unique design, versatility, safety, ease of production, ease of handling, absence of any type of microorganism, etc. (Foroutan and Ghaffarifar, 2018[15]; Li and Petrovsky, 2016[33]). Accumulating evidence has been shown that successful DNA immunization tends to stimulate Th1-type rather than Th2-type immune response (Li and Petrovsky, 2016[33]). There are different ways of administering a DNA vaccine to the body, including syringe injection (intramuscular, subcutaneous, mucosal), gene gun immunization, nanoparticles, liposomes, oral delivery pathway or nasal spray (Doria-Rose and Haigwood, 2003[7]; Li and Petrovsky, 2016[33]). In general, direct injection of naked plasmid DNA enters to the cell cytoplasm which can express encoded proteins, thereby enhancing specific humoral and cell-mediated immune responses (Li and Petrovsky, 2016[33]). Despite the advances in DNA vaccine technology, there are various limitations associated with the use of DNA vaccines, which occasionally confined the immunogenicity of them such as dosages of inoculum, the delivery route, and also inadequate ability to produce non-protein antigens (Foroutan and Ghaffarifar, 2018[15]; Li and Petrovsky, 2016[33]). In order to provide sufficient protection and prevent from the attachment of *T. gondii *to related host cell receptors, B-cell activation and antibody production are needed. In this regard, Immunoglobulin G (IgG) as an essential antibody can eliminate the parasite via activation of the classical complement cascade as well as enhancement of killing activity of macrophages (MQs) (Sayles et al., 2000[42]). Furthermore, the production of nitric oxide (NO) from activated MQs is able to control *T. gondii* replication (Cabral et al., 2018[5]). 

Various studies have shown that protection against *T. gondii* infection is developed through both humoral and cellular immune responses as well as regulatory cytokines. Generally, secretion of interferon-γ (IFN-γ) from CD_4_^+^ and CD_8_^+^ T cells to be effective at controlling and limiting parasite growth. Also, this signature cytokine could inhibit reactivation of intracellular bradyzoites during the chronic phase of infection (Denkers and Gazzinelli, 1998[6]; Suzuki et al., 1988[46]). In addition, interleukin 2 (IL-2) and IL-12 secreted through Th1 type cells can promote host resistance against *T. gondii* infection, whereas type 2 cytokines such as IL-4, IL-5, IL-9, IL-10, and IL-13 regulates Th2 responses (Mosmann and Moore, 1991[35]; Zhang et al., 2015[50]). The studies have shown that good DNA vaccines stimulate major histocompatibility complex (MHC) class I and II molecules that lead to the activation of CD_8_^+^ and CD_4_^+^ T-cell immune responses against this opportunistic agent (Li and Petrovsky, 2016[33]; Tighe et al., 1998[47]). 

Recently, it was shown that DNA vaccination with ROM1, ROM4 and ROM5 has become popular and can promote an appropriate immune response against *T. gondii* infection (Han et al., 2017[24]; Li et al., 2012[32]; Rahimi et al., 2017[38]; Zhang et al., 2015[51]). In fact, calcium phosphate nanoparticles (CaPNs) are considered as adjuvants or delivery vehicles that extend the antigen releasing period to evoke strong and long-lasting immune responses with increasing the uptake of antigen by antigen presenting cells (APCs) (Gregory et al., 2013[23]). In this context, Rahimi et al. (2017[38]) designed an experimental study to evaluate the protective efficacy of ROM4 alone or with coated CaPNs as the adjuvant in BALB/c mice. They reported that co-administration of CaPNs with pcROM4 increases the survival time of the vaccinated groups (*P* < 0.05) than those mice in control groups when challenged with 1×10^3^ tachyzoites of RH strain. Furthermore, a mixed Th1/Th2 response with the predominance of IgG2a over IgG1 (*P *< 0.05), and high secretion of IFN-γ (*P *< 0.05) were observed as the outcome of immunization, compared with control groups. This data suggests that immunization with nanoadjuvant of CaPNs as a novel adjuvant for DNA vaccine could elicit a strong specific immune response (Rahimi et al., 2017[38]). In another study, Zhang et al. (2015[51]) designed a comprehensive study on ROM4 and ROM5 proteins against acute and chronic *T. gondii* infection in Kunming mice. The mice immunized with pVAX-TgROM5 or pVAX-TgROM4 revealed higher levels of some cytokines, as well as increased the level of IgG antibody titers (the predominance of IgG2a production). Notably, the percentage of CD_4_^+^ and CD_8_^+^ T cells were enhanced (*P* < 0.001). Besides, the number of brain cysts were significantly reduced (72.04 % for pVAX-TgROM5 and 44.08 % for pVAX-TgROM4, respectively) and increased survival rate (20 % and 13.3 %, 35 days post challenge in mice immunized with pVAX-TgROM5 and pVAX-TgROM4, respectively) compared with control groups (death within 8 days, *P* < 0.05). These results indicated that pVAX-TgROM5 is to be more effective than pVAX-TgROM4 that can enhance the protective immunity against acute and chronic *T. gondii* infection (Zhang et al., 2015[51]). TgROM1 composed of adhesive proteins that is located in the secretory pathway of the parasite. It is well known that it is essential for parasite growth *in vitro* as well as expressed in the tachyzoite stage of *T. gondii* (Brossier et al., 2008[3]). Li et al. (2012[32]) evaluated the protective efficacy of TgROM1. The findings showed that pVAX-ROM1 is able to induce the high production of IFN-γ, IL-2, IL-4, and IL-10 significantly compared with those groups that injected pVAX1 alone or PBS (*P* < 0.05). Moreover, higher percentages of T-CD_4_^+^ cells (*P* < 0.05) and higher levels of IgG antibodies than control (*P* < 0.05) were recorded. Also, pVAX-ROM1 immunization, leads to higher survival time (12.5 ± 0.7 days, *P* < 0.05) than those mice in control groups. The authors suggested that TgROM1 could be considered as a promising vaccine candidate against *T. gondii* infection (Li et al., 2012[32]). More details of immunization experiments with DNA vaccines against *T. gondii* infection are listed in Table 2(Tab. 2) (References in Table 2: Han et al., 2017[24]; Li et al., 2012[32]; Rahimi et al., 2017[38]; Zhang et al., 2015[51]). 

## ROM Peptide Vaccine

In recent decade, bioinformatics as a newfound interdisciplinary science has become popular among scientists which analyze the biological data by recruiting the defined technologies and algorithms from statistics, physics, computer sciences, mathematics, medicine, and biology (Romano et al., 2011[39]). This novel science can be used for several purposes, including predicting protein structures, biological characteristics, functions, epitopes, design of new vaccines, etc. It is worth to mention that bioinformatics techniques had satisfactory precision and accuracy and needed low time (Foroutan et al., 2018[17]; Khademvatan et al., 2013[27]; Romano et al., 2011[39]; Wang et al., 2016[48]). The prediction of potent epitopes is necessary to evaluate the immunogenicity of target antigen for design of reverse vaccines. Hence, bioinformatics tools and online servers surely assist researchers to predict and identify the potential B and T cell epitopes (Foroutan et al., 2018[17]; Wang et al., 2016[48]).

In this context, Han et al. (2017[24]) conducted a comprehensive study. In brief, the antigenic features of ROM4 and SAG1 were analyzed and then compared together by employing bioinformatics tools. The DNASTAR outputs revealed that ROM4 had a better antigenic index, surface probability, and flexibility than SAG1 molecule. Moreover, the IC_50_ values of HLA-DRB1*01:01, H2-IAb, H2-IAd, and H2-IEd alleles of ROM4 were smaller than those of SAG1, indicating that ROM4 may have better Th epitopes than SAG1. The authors identified a polypeptide (YALLGALIPYCVEYWKSIPR) using bioinformatics methods and then were tested in BALB/c mice. After immunization, increased levels of IgG antibody response were observed. Also, in subsets of IgG, the predominance of IgG2a over IgG1 was recorded. In those mice that vaccinated with ROM4 peptide, the production of IFN-γ, IL-2, and IL-12 were significantly increased, compared with control groups. Also, increased survival time (*P* < 0.05) and reduction of brain cysts load (*P* < 0.05) were seen, compared with control groups (Table 2(Tab. 2)). The authors remarked that this vaccine could be considered as a potent promising vaccine candidate against chronic and acute *T. gondii* infection (Han et al., 2017[24]).

## Prime-Boost Strategies

Traditional vaccines such as live attenuated micro-organisms and inactivated micro-organisms are used in medical purposes widely, but these vaccines are not any more appropriate choices because of several reasons, including lack of adequate efficiency, etc. (Lee and Nguyen, 2015[31]). As evident, subunit vaccines are based on peptides, proteins or polysaccharides containing protective antigens. However, the recombinant subunit vaccines are poorly immunogenic and to solve this problem, usually require some additional components to elicit the immune response. Hence, the use of some adjuvants and also repeated boost immunizations are suggested to elevate the efficiency of subunit vaccines (Hansson et al., 2000[25]; Kardani et al., 2016[26]; Lee and Nguyen, 2015[31]). Subunit vaccines mainly elicit a humoral immune response, while recombinant live vector vaccines and DNA vaccines predominantly induce the cellular immunity (Kardani et al., 2016[26]; Nascimento and Leite, 2012[36]). One way to overcome this shortage is prime-boost strategy which is a perfect technique to improve the efficiency of vaccination (Kardani et al., 2016[26]). In fact, the prime-boost strategy generates a more powerful immune response and elicit both humoral and cellular immune responses (Han et al., 2017[24]; Li and Petrovsky, 2016[33]). The mechanism of prime-boost methods is as follows: the initial immunization primes the immune response and following immunizations motivate extra expansion of antigens (Kardani et al., 2016[26]; Li and Petrovsky, 2016[33]). One key issue in prime-boost immunization is the order of vaccination process, e.g. sequence of using DNA as the prime and protein as the boost, the other important factor is the interval between using prime and boost in vaccination procedure, considering these aspects of prime-boosting immunization lead to strong immune responses (Foroutan et al., 2018[17]; Kardani et al., 2016[26]).

The prime-boost method consists of two types: homologous and heterologous prime-boost regimens. Homologous prime-boost regimen covers the same formulation used in both prime and boost regimens, while heterologous prime-boost regimens involve different formulations (Kardani et al., 2016[26]; Li and Petrovsky, 2016[33]). Based on several studies, heterologous prime-boost immunizations have proven to be appropriate ways for immunization against different infections (Kardani et al., 2016[26]; Li and Petrovsky, 2016[33]). Since, heterologous prime-boost regimens strongly induce both humoral and cellular immune responses and elicits a robust immune response, it is more efficient and more potent than homologous prime-boost (Kardani et al., 2016[26]). Boosting a primary response with a heterologous vector leads to 4 to 10-fold higher responses in comparison with homologous prime-boost method (Kardani et al., 2016[26]). Heterologous prime-boost strategies principally use a DNA or a viral vector, particularly adenovirus for priming and a protein for boosting (Li and Petrovsky, 2016[33]), using various vectors leads to improved levels of CD_4_^+^ and CD_8_^+^ T-cells in contrast to homologous boosting (Dunachie and Hill, 2003[13]).

In this case, Han and colleagues (2017[24]) used DNA-priming and polypeptide-boosting regimen based on ROM4 (pROM4 prime/ ROM4 peptide boost) in BALB/c mice in order to investigate the levels of IgG, IgG2a, IgG1, and the main cytokines in different mouse groups. The results indicate that BALB/c mice vaccinated with DNA/peptide had the most levels of the IgG, IgG2a, IgG1, IL-2, IL-12 and IFN-γ. Moreover, the mice vaccinated with DNA/peptide showed the maximum survival time and the lowest brain cyst load in comparison with the control group. About 1×10^4^ tachyzoites of *T. gondii* RH strain were intraperitoneally injected to all mice. All mice in the negative control groups died on day 6, but those groups which received prime-boost regimen, survived until day 18. The authors concluded that ROM4 induces the highest level of immune response than other regimens, which could be considered as a promising approach to elevate the efficacy of vaccination against acute and chronic *T. gondii* infection (Han et al., 2017[24]).

## Concluding Remarks

*T. gondii* infects several intermediate hosts such as rodents, birds, marine mammals, domestic/wild mammals, humans, etc. For example, approximately one third of the total individuals around the globe have anti-*T. gondii* antibodies in their sera. In the two past decades, an increasing number of papers have been published using different vaccine types and various strategies to assess the immunogenicity of *T. gondii* antigens. However, these attempts failed to introduce an effective vaccine against *T. gondii* infection, but promising achievements were obtained by researchers. The use of DNA vaccines encoding ROM1, ROM4, and ROM5 alone or in combination with other antigens elicited the immune responses, prolonged the survival time/rate and reduced the brain cysts load among vaccinated mice. In addition, the use of prime-boost regimen by recruiting ROM4 increased the survival duration up to 18 days post challenge. Nevertheless, these reports failed to show complete protection in immunized mice. It should be noted that ROM4 is expressed in the majority stages of *T. gondii* life cycle (primarily in tachyzoites, at lower levels in bradyzoites, and weakly in sporozoites). Also, it is involved in several vital functions of *Toxoplasma*. So that, the suppression of ROM4 leads to the following defects: partially impairment in lytic growth, reduction of the frequency of moving junction formation, and disruption of normal gliding. In regard to the above mentioned points, the vast use of ROM4 by researchers is justifiable. However, there are no published articles about ROM2, ROM3, and ROM6 in terms of vaccination against *T. gondii* infection. On the other hands, unfortunately, there were no reports regarding the use of vaccines based on live-attenuated vectors and multi-epitope vaccines for ROM-based antigens, which require more consideration in future investigations by scientists. Moreover, co-delivery of genetic and/or non-genetic adjuvants surely would influence the outcomes in vaccinated groups.

## Notes

Masoud Foroutan and Leila Zaki contributed equally as first authors.

## Acknowledgements

The authors would like to thank all staff of Abadan School of Medical Sciences, Iran.

## Ethics approval and consent to participate

Not applicable.

## Conflict of interest

The authors declare no potential conflicts of interest with respect to the research, authorship, and/or publication of this article.

## Figures and Tables

**Table 1:**
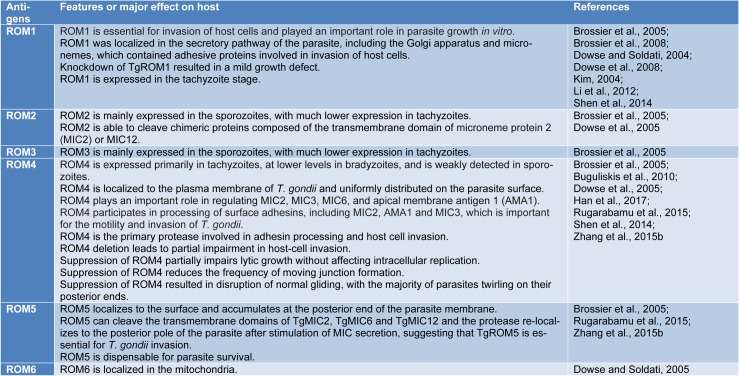
The main features and functions of some ROMs

**Table 2:**
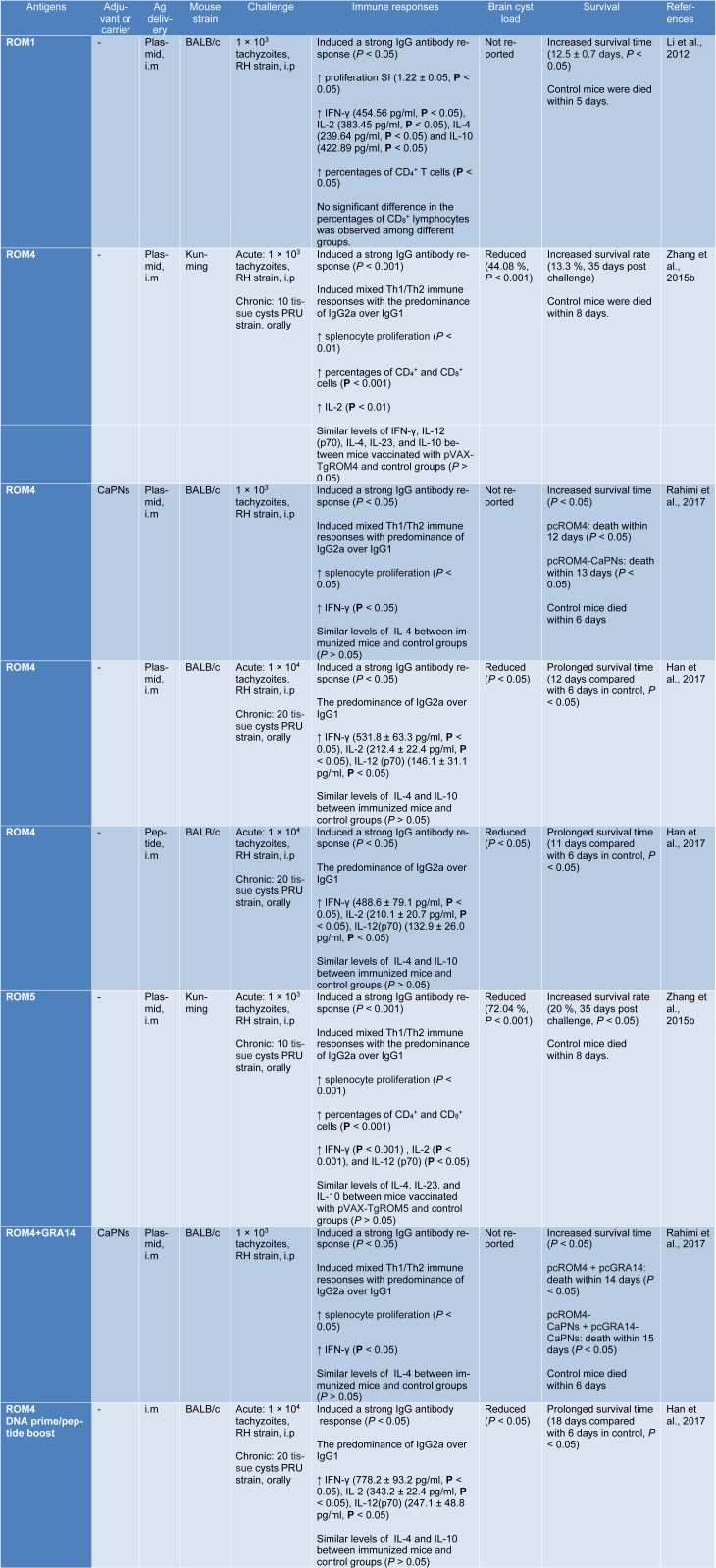
Baseline characteristics of included studies
